# Strategies to Uplift Novel Mendelian Gene Discovery for Improved Clinical Outcomes

**DOI:** 10.3389/fgene.2021.674295

**Published:** 2021-06-17

**Authors:** Eleanor G. Seaby, Heidi L. Rehm, Anne O’Donnell-Luria

**Affiliations:** ^1^Program in Medical and Population Genetics, Broad Institute of MIT and Harvard, Cambridge, MA, United States; ^2^Genomic Informatics Group, University Hospital Southampton, Southampton, United Kingdom; ^3^Center for Genomic Medicine, Analytic and Translational Genetics Unit, Massachusetts General Hospital, Boston, MA, United States; ^4^Division of Genetics and Genomics, Boston Children’s Hospital, Boston, MA, United States; ^5^Manton Center for Orphan Disease Research, Boston Children’s Hospital, Boston, MA, United States

**Keywords:** Mendelian, novel gene discovery, disease–gene relationships, rare genetic disorders, genomics, rare disease, genetics

## Abstract

Rare genetic disorders, while individually rare, are collectively common. They represent some of the most severe disorders affecting patients worldwide with significant morbidity and mortality. Over the last decade, advances in genomic methods have significantly uplifted diagnostic rates for patients and facilitated novel and targeted therapies. However, many patients with rare genetic disorders still remain undiagnosed as the genetic etiology of only a proportion of Mendelian conditions has been discovered to date. This article explores existing strategies to identify novel Mendelian genes and how these discoveries impact clinical care and therapeutics. We discuss the importance of data sharing, phenotype-driven approaches, patient-led approaches, utilization of large-scale genomic sequencing projects, constraint-based methods, integration of multi-omics data, and gene-to-patient methods. We further consider the health economic advantages of novel gene discovery and speculate on potential future methods for improved clinical outcomes.

## Introduction

Rare genetic disorders affect 1-in-17 individuals in their lifetime (Turnbull et al., 2018). They encompass some of the most severe disorders affecting patients worldwide, including childhood cancers, neurodevelopmental disorders, and muscle diseases to name a few (Simpson, 2016). Many are severe and life limiting, with significant morbidity and mortality. Indeed, 30% of children with rare diseases die before their fifth birthdays (Rode, 2005). Many affected patients are wheelchair bound and require respiratory support, feeding support, specialized community services, and significant hospitalizations (Yoon et al., 1997; Dodge et al., 2011). This not only impacts the patients involved but their caregivers and families as well.

Approximately 80% of rare diseases have a genetic basis, yet many of the underlying genes have not yet been identified, nor has the wide spectrum of pathogenic variation been delineated for each gene (Yoon et al., 1997; Dodge et al., 2011; Wright et al., 2018). As such, on average across all specialties, the causal variant(s) are only identified in ∼30–40% of rare disease patients, leaving the majority of patients and their families without a reliable prognosis, rendering medical care largely supportive and palliative (Retterer et al., 2016; Adams and Eng, 2018; Clark et al., 2018; Srivastava et al., 2019).

One of the biggest challenges in reaching a molecular diagnosis is the paucity of scientific knowledge into the biological function of all ∼20,000 human genes. Indeed, the disease phenotypes have yet to be discovered for ∼50% of those genes with a genomic signature suggesting haploinsufficiency (Seaby and Ennis, 2020). Therefore, diagnosing rare diseases is extremely challenging without a prior correlation between a clinical phenotype and causative gene. New gene disorders preclude detection when for every genome sequenced, millions of variants of uncertain significance reside in genes of unknown function (Smedley et al., 2015; Karczewski et al., 2020). Even the best computational methods available at present will typically overlook a gene of undetermined biological significance when analyzing a patient’s exome (restricted to protein-coding regions of DNA) or genome (all DNA regions). Therefore, new rare genetic diseases will be overlooked until further studies are undertaken or new methods are developed to uplift novel gene discovery (Seaby and Ennis, 2020).

## Why Is Novel Mendelian Gene Discovery Important?

The significance of uplifting novel Mendelian gene discovery is not to be underestimated. Every new disease gene discovered goes toward ending the notorious “diagnostic odyssey” of rare disease. This pertains to rare disease patients who move between specialties and undergo myriad diagnostic tests in search for a unifying genetic explanation (Thevenon et al., 2016). For most, these often expensive evaluations only elucidate the clinical phenotype and seldom aid in diagnosis. In the United Kingdom, over a 10-year period, undiagnosed rare diseases have cost NHS England an average of £13,064 (US$18,279) per patient and in excess of £3.4 billion (US$4.8 billion) in total (Imperial College Health Partners, 2018). In Australia, the cost per diagnosis using standard care is AU$27,050 (US$21,241), and in the United States, the same cost basis was calculated at US$19,100 (Soden et al., 2014; Stark et al., 2017). While these figures all showcase the cost burden of rare diseases, it is ill advised to compare cost evaluations between countries due to differing healthcare systems.

Novel discoveries directly impact diagnostic potential. Diagnoses not only provide answers for patients and families but have far-reaching clinical impact, including but not limited to guiding personalized treatments; offering patient support networks; collecting and gaining knowledge on disease trajectory and prognosis; enabling participation in research studies; informing reproductive choices; and impacting the health of relatives. Even when little can be done therapeutically following diagnosis, the importance of that diagnosis to patients and families should not be overlooked; when a cause is identified, this often alleviates guilt and blame felt by patients and families who believe a given rare disease is their fault (Muir, 2016).

Novel gene discovery is critical in the research space to expand biological understanding of human genes and variation and to identify therapeutic drug targets that may lead to successful and life-altering therapies (Legendre et al., 2013; Ribeil et al., 2017; Mirmiran et al., 2019). Gene augmentation therapies have been developed for a number of conditions, for example, subretinal injection of adeno-associated virus vectors to deliver *RPE65* cDNA to treat Leber congenital amaurosis (MIM: 204100) (Maguire et al., 2009; Pierce and Bennett, 2015) and the FDA-approved one-time intravenous administration of *SMN* cDNA to treat spinal muscular atrophy type 1 (MIM: 253300) (Mendell et al., 2017; Beck et al., 2020). Small molecular therapies for cystic fibrosis (MIM: 602421) are well studied and include ivacaftor, which increases the time fraction that the cystic fibrosis transmembrane conductance regulator (CFTR) channel remains open, and lumacaftor, which increases the amount of CFTR that reaches the cell surface (Wainwright et al., 2015; Habib et al., 2019). Development of antisense oligonucleotides is proving effective in preclinical and clinical studies to treat neurodegenerative diseases (Smith et al., 2006; Ly and Miller, 2018); and it is hoped that identification of novel disease genes may guide further protein targets.

In contrast to the development of new gene therapies, it is not uncommon for existing therapies to be repurposed when knowledge of a given gene and biological pathway is implicated in diseases. For example, in 2011, autosomal recessive variants in *MTHFD1* (a gene involved in folate metabolism) were found to cause combined immunodeficiency and megaloblastic anemia with or without hyperhomocysteinemia (MIM: 617780) (Watkins et al., 2011). Simple folic acid has proven life changing for patients with recessive mutations in *MTHFD1* (Burda et al., 2015; Ramakrishnan et al., 2016).

## The Prior Decade of Novel Gene Discovery

Since the advent of next-generation sequencing (NGS) technologies, there has been a stepwise acceleration in novel gene discovery leading to uplifted diagnostic rates for rare disease patients (Chong et al., 2015; Posey et al., 2019). Between 2005 and 2009, there were ∼170 novel discoveries per year. This is compared to ∼240 per year between 2010 and 2014 when NGS became widely adopted (Chong et al., 2015). In the history of disease–gene relationship discovery, NGS approaches are responsible for ∼36% of all reported Mendelian disease genes. Their contribution to novel gene discoveries is accelerating, with 87% of new gene disorders now discovered using NGS approaches (Bamshad et al., 2019). Novel discoveries are still progressing, although the pace of discovery appears to have reached a steady state that balances the time required to build international cohorts, undertake functional experiments, and publish findings (Posey et al., 2019). Despite this, approximately 250 new genes are added to the literature annually, and a recent review predicted that more than 6,000 Mendelian conditions remain to be discovered (Bamshad et al., 2019). Therefore, with thousands of monogenic disease–gene relationships yet to be elucidated, there is clear evidence that the recognition of disease-causing variation in the exome is far from saturated (Posey et al., 2019). For more data on the pace of novel discoveries, we recommend the excellent review by Bamshad et al. (2019).

## Traditional FamiLy-Based Approaches to Diagnosis and Novel Gene Discovery

At present, most exome and genome analyses are conducted on a “per family” basis, that is, to say on a small number of related individuals, most commonly a trio (parents and child). Analyzing NGS data is labor-intensive, taking up to 20–40 h to compile a report (Dewey et al., 2014). The challenge of handling vast quantities of genomic data has improved with advancing methods; however, for each exome or genome sequenced (and depending on whether segregation analysis is available using family studies), there are anywhere from tens to thousands of plausible candidate variants (Adams and Eng, 2018). If a variant is found in a known disease gene, a rapid diagnosis can often be made with rigorous variant curation against laboratory standard guidelines (Richards et al., 2015; Amendola et al., 2016). However, for ∼60% of rare disease patients who undergo clinical exome or genome sequencing, their sequencing report is non-diagnostic, despite the fact that for many, the causal variant is present but unrecognized in their sequencing results. New diagnoses therefore cannot be made until new disease–gene relationships are discovered and the full spectra of pathogenic or disease-causing variants in known disease genes are elucidated (Seaby and Ennis, 2020).

It is worth noting that there is a difference between clinical testing and research-led sequencing studies. Clinical testing typically focuses on variants in established or known disease genes, whereas research studies have scope to evaluate variants in genes of unknown function or not currently linked to disease. Unless patients undergoing diagnostic testing are additionally recruited into research studies, opportunities to evaluate variants in new disease genes are limited. That said, there is increasing involvement of diagnostic centers with research laboratories and affiliated universities; indeed, many families are now concomitantly offered diagnostic testing and recruitment to further research studies. However, for these diagnostic labs capable of bridging the gap between clinical testing and research, many are ill-equipped to investigate the plethora of plausible disease candidates remaining after variant filtration and prioritization. Theoretically, the only way to establish new diagnoses when novel genes are identified in a research setting is to conduct functional experiments on all potential candidates. This approach is proving a major bottleneck as most research laboratories do not have the finances, nor the time or resources, to support functionally validating a large number of candidate variants without any guarantee that the selected variants are disease causing. Few laboratories will invest resources into a particular variant for one patient without additional kindreds with overlapping phenotypes or prior published studies on the gene’s function. Many current practices in gene discovery are still hindered by a “bottom-up” approach that takes single-patient cases that brings the phenotype to a novel gene; a piecemeal approach limited by intensive functional experiments on genes of equally predicted causality. Therefore, efforts in recent years have focused on strategies to prioritize the best candidates for functional follow-up.

## Strategies to Uplift Novel Gene Discovery

### Collaborative, Data-Sharing Approaches

Collaborative projects, data sharing, and building disease cohorts have proven invaluable in genomics. In 2010, *MLL2* (*KMT2D*) was discovered as the cause of the Kabuki syndrome (MIM: 147920). Ten unrelated patients with the same characteristic clinical phenotype underwent exome sequencing. Seven of the 10 individuals were found to have loss-of-function variants in *MLL2*, which led to its disease association (Ng et al., 2010). Historically, the approach of building a case series of affected individuals has been a rate-limiting step, relying on local connections or collaborations built through conferences or publications. Given the rarity of monogenic disorders, it can take many years to accrue sufficiently sized cohorts with similar clinical features and genotypes. This method is therefore inefficient and inadequate to rapidly support novel gene discovery (Azzariti and Hamosh, 2020).

In 2017, the directors’ board of the American College of Medical Genetics and Genomics released a position statement on how genomic data sharing is critical to improving genetic healthcare (ACMG Board of Directors, 2017). With an ever more connected world, global efforts to share genotype and phenotype data have proven essential in the endeavor of novel gene discovery. Improved data governance, drives for open data science, and advancing informatics methods have since led to the practice of genomic matchmaking, facilitating researchers and clinicians from across the globe to share phenotype/genotype data for accelerated discoveries (Azzariti and Hamosh, 2020).

### Matchmaker Exchange

**FIGURE 1:**
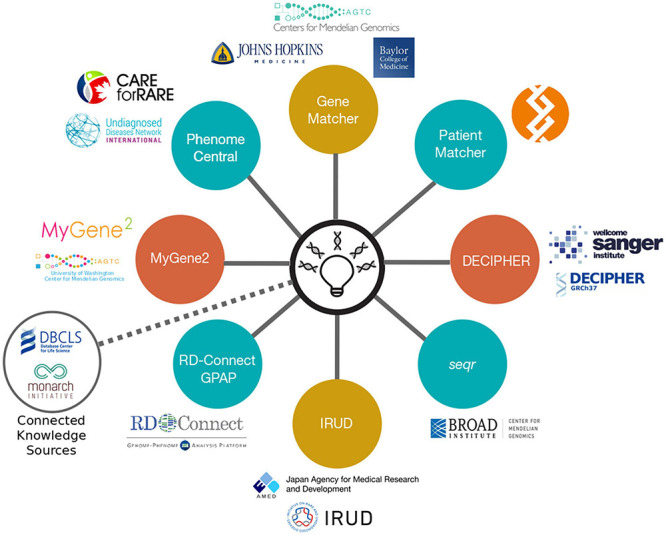
The MME API and its connected nodes. MME uses a federated network of nine connected nodes. Image taken from https://www.matchmakerexchange.org/.

In 2015, the Matchmaker Exchange (MME) was launched, providing a systematic and robust approach to novel Mendelian gene discovery by facilitating a mechanism for matching patients across genomic centers, research laboratories, diagnostic laboratories, and physicians through a federated network (Figure 1) (Philippakis et al., 2015).

MME builds on the success of earlier genomic matchmaking platforms by connecting datasets through an application programming interface (API) enabling searches of multiple databases with a single query. The advantage of using a federated network enables individual submitters to maintain control and autonomy over their data and keep the content up to date, while ensuring compliance with their local and national data-sharing policies (Azzariti and Hamosh, 2020). By identifying additional affected kindreds with overlapping phenotypes, the best candidate variants and genes can be targeted for functional validation. The MME API has been widely adopted by scientists and clinicians globally and has led to numerous international collaborations and publications. One such example is the discovery of a KMT2E-related neurodevelopmental disorder, the O’Donnell-Luria-Rodan syndrome (MIM: 618512), following identification of 38 individuals from 36 families, of which 28 were ascertained using MME. This discovery goes beyond elucidating disease–gene etiology and has identified a potential therapy already widely used in healthcare that could be evaluated for this syndrome (O’Donnell-Luria et al., 2019).

### Patient-Led Approaches

It could be argued that no one is more invested in ending the diagnostic odyssey of rare disease than affected individuals and their families. In an era when patients are actively involved in research studies as participants (Kaye et al., 2012), it is unsurprising that patients and caregivers are also invested in genomic matchmaking efforts (Lambertson et al., 2015). Patients and families are beginning to take control of their own data and utilize open data sharing and social media in an effort to discover new genetic disorders. Embedded within the MME API is a family-facing platform called MyGene2, which gives patients and caregivers autonomy over their data, facilitating direct data sharing when desired, while still enabling scientists and clinicians to access these shared anonymized data (Azzariti and Hamosh, 2020).

Social networking sites such as Facebook, Twitter, and Instagram are also proving popular with patients/caregivers as a matchmaking resource (Macnamara et al., 2019). In 2014, Matthew and Cristina Might harnessed the power of social media to identify additional cases of NGLY1 deficiency, leading to identification of a new gene disorder (Might and Wilsey, 2014; Might and Might, 2017). Following their son’s diagnosis, the Might family explored options for conceiving a child unaffected by the same condition. Their son’s diagnosis facilitated not only conception of a healthy sibling but a pathway for other affected families to conceive healthy children using preimplantation genetic testing or non-invasive prenatal diagnostic testing (Might and Might, 2017). The Might family created a legacy for others to follow, having built a global community of families providing mutual support, in addition to facilitating research and international *NGLY1* meetings (Might and Wilsey, 2014).

Inspired by the success of the Might family, families across the globe have harnessed the networking potential of social media to match with other affected kindreds with similar phenotypes and genotypes. Indeed, social media additionally facilitated the identification of three children with variants of uncertain significance in *KDM1A*, leading to discovery of another novel gene disorder (MIM: 616728) (Chong et al., 2016). The success of such endeavors has now inspired the Undiagnosed Diseases Network (UDN), started at the National Institute for Health in 2008, with 11 additional clinical sites across the United States, to use social media in a similar way. With appropriate consent, webpages are created for individual participants, showcasing the clinical phenotype, significant variants, and candidate genes. This approach has proven successful in identifying additional affected patients with variants in *NACC1*, leading to the discovery of its associated phenotype (Macnamara et al., 2019).

### Large-Scale Programs for Novel Gene Discovery

Genomic sequencing has become increasingly affordable and possible in recent years. However, exome and genome sequencing are seldom first-line investigations, with many healthcare systems and health insurance policies not covering the cost. This has perhaps inspired the creation of large-scale international sequencing programs, often with government funding, offering exome and/or genome sequencing to thousands of rare disease patients and their families. These projects benefit from pooled resources and focus on diagnosing patients who were undiagnosed following conventional clinical testing, in addition to better elucidating the underlying mechanisms of Mendelian diseases. Such examples include the United Kingdom’s 100,000 Genomes Project (100KGP) (Turnbull et al., 2018) and Deciphering Developmental Disorders study (Firth et al., 2011) as well as the United States’ Centers for Mendelian Genomics (Bamshad et al., 2012). These programs benefit from sequencing large numbers of patients with improved power to match patients with similar genotypes and phenotypes, both internally and through the MME (Philippakis et al., 2015). Furthermore, most of these programs recruit patients for both clinical diagnostics and follow-on research, meaning that where possible, novel discoveries and variants of uncertain significance can be investigated further; this has previously been a limitation of clinical diagnostic studies (Seaby and Ennis, 2020). Consequently, thousands of novel gene discoveries have been identified through these projects. Indeed, by 2018, the Centers of Mendelian Genomics had reported >3,500 disease gene–phenotype pairs, expanding both known and novel disease gene associations (Posey et al., 2019). And in 2020, 28 new genetic disorders were discovered by leveraging data from the Deciphering Developmental Disorders study (Kaplanis et al., 2020).

### Phenotype-Driven Approaches

In recent years, novel gene discovery has shifted from phenotype-driven methods to genotype-driven approaches, i.e., taking genotype data and matching phenotypes to that genotype through matchmaking efforts, though both remain important (Bamshad et al., 2019). Efforts to standardize phenotype terms through the Human Phenotype Ontology (HPO) database have aided comparative statistics using a universal library of agreed clinical terms involved in disease (Robinson et al., 2008). This has paved the way for computational phenotype analyses that can assess a candidate gene’s relevance to phenotype data observed in patient(s). A number of tools (Table 1) have been developed that estimate the similarity between HPO terms in an individual and those representing disease in a database. By incorporating phenotype ontology data across species, these tools are capable of prioritizing candidate genes without known disease association (Köhler et al., 2009; Schulz et al., 2011; Bauer et al., 2012). Similar approaches have been commercialized, taking advantage of advanced artificial intelligence to identify and rank potential disease-causing variants following a multidimensional analysis; examples include Fabric Genomics^1^ and Emedgene^2^. More experience and data are needed to understand the strengths and limitations of these tools.

**TABLE 1:** Four phenotype-driven tools for prioritization of known and novel disease genes.

**Tool**	**Principle**	**Application**	**Access**
Exomiser (Smedley et al., 2015)	Uses random-walk analysis of protein–protein interaction networks, cross-species phenotype comparisons, and a wide range of additional filters that consider prediction models, disease segregation, and allele frequency	Focused on identifying novel and known disease genes	http://www.sanger.ac.uk/science/tools/exomiser
eXtasy (Sifrim et al., 2013)	Prioritizes non-synonymous variants predicted to be pathogenic using a fusion methodology that integrates multiple strategies in a phenotype-specific manner	Focused on identifying candidates in novel and known disease genes	http://extasy.esat.kuleuven.be/
Phevor (Singleton et al., 2014)	Combines outputs of multiple biomedical ontologies and propagates patient phenotype information across and between ontologies for improved variant interpretation	Focused on identifying candidates in novel and known disease genes	http://weatherby.genetics.utah.edu/cgi-bin/Phevor/PhevorWeb.html
Phen-Gen (Javed et al., 2014)	Uses a systematic Bayesian framework which combines patient sequencing data with phenotype information for improved rare disease variant analysis of both coding and non-coding variation	Focused on identifying candidates in novel and known disease genes	http://phen-gen.org/

*Bioinformatic phenotype-driven tools for the detection and prioritization of variants in novel and known disease genes using the integration of patient genotype and phenotype data.*

One of the challenges for novel gene discovery is the requirement for accurate and deep phenotyping. Optimally, this should be collected longitudinally (Seaby and Ennis, 2020). While HPO terms do help to standardize the recording of phenotype information and indeed are used universally in many databases including those connected through MME (Philippakis et al., 2015), they are often only collected at a point in time and may lack the “full narrative” of the clinical history. This can be problematic when assessing new genotype–phenotype correlations, since for many neurodevelopmental disorders, phenotypes can significantly overlap. It can also be difficult to weight the severity or prominence of clinical features as the conversion to a list of terms tends to weight all the features similarly.

### Constraint-Based Approaches

Given the mutation rate and the Earth’s current population size, we expect to observe every variant compatible with life in a living human. Indeed, the aggregation of large population datasets has begun to reveal the spectrum of damaging variants across the human genome (Lek et al., 2016; Karczewski et al., 2020). It is typical to observe approximately 100 loss-of-function variants per genome with ∼20 genes completely inactivated (knockouts) even in perfectly healthy individuals from the general population (MacArthur et al., 2012). Population data can be utilized to evaluate the strength of natural selection at the gene level and to differentiate rare from common loss-of-function variants. As deleterious variants are purged from human populations through natural selection, there are opportunities to identify genes and regions that are constrained for variation compared to expected mutation rates, revealing which genes are most intolerant to inactivation of one (haploinsufficient) or both (knockout) copies (Samocha et al., 2014; Karczewski et al., 2020).

### Loss-of-Function Constraint

**FIGURE 2:**
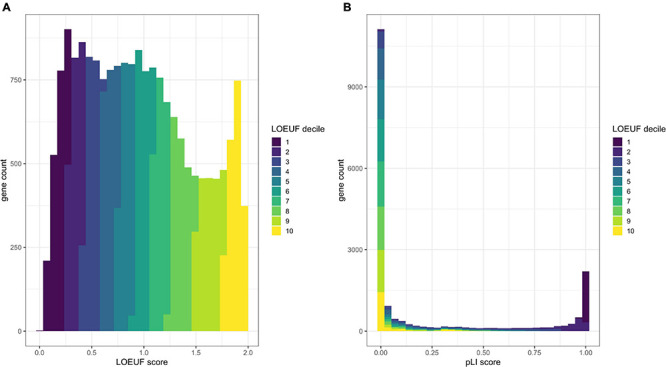
Comparison of the distribution of pLI and LOEUF. Panel **(A)** shows a histogram of human genes across the LOEUF spectrum displaying a continuous pattern. Lower scores represent higher gene constraint (for loss of function). The histogram is colored by the LOEUF decile. Panel **(B)** shows a histogram of human genes across the pLI spectrum. This spectrum is extremely dichotomous with the majority of genes skewed toward either 0 (not constrained for loss of function) or 1 (constrained for loss of function). This can help to discriminate genes that are likely to cause disease through haploinsufficiency (pLI > 0.9). The dichotomous nature of pLI is by design, as initially the reference databases were too small to have adequate power to discern depletion for loss-of-function variation in small- to medium-length genes. The pLI distribution is colored by the LOEUF decile to show the overlap between scores. Higher pLI scores correlate with lower LOEUF scores as expected. The continuous nature of the LOEUF score provides more granular detail than pLI across the middle of the spectrum and can better stratify genes with moderate levels of constraint that may be implicated in recessive disease.

Lek et al. (2016) (Kosmicki et al., 2017) defined a set of genes with high probability of intolerance to heterozygous predicted loss-of-function variation (pLI) modeled on ∼60,000 exomes from the Exome Aggregation Consortium (ExAC) population database (Lek et al., 2016). This pLI score can be used to identify candidate haploinsufficient disease genes constrained for loss of function in a dichotomous way; i.e., a gene is predicted to be haploinsufficient (pLI > 0.9) or not (Figure 2).

Karczewski et al. (2020) refined the model and regenerated pLI scores utilizing a larger dataset of ∼141,000 exomes and genomes from the Genome Aggregation Database (gnomAD)^3^. The authors also developed the Lower Observed/Expected Upper-bound Fraction (LOEUF) score, a continuous metric which places >19,000 human genes on a spectrum of intolerance to inactivation (Figure 2). Genes with the lowest LOEUF scores, i.e., the fewest loss-of-function variants compared to an expectation, are the most constrained for loss of function, highlighting their potential biological essentiality. Both LOEUF and pLI were validated by comparison to several orthogonal indicators of constraint and shown to be accurate at discriminating haploinsufficient disease genes from autosomal recessive and polymorphic (unconstrained) genes (Karczewski et al., 2020). A companion paper by Collins et al. (2020) additionally showed that structural variants share the same pattern of constraint as LOEUF and are responsible for about a quarter of all rare loss-of-function events per genome.

As LOEUF identifies genes constrained for loss-of-function variation, we expect these genes to be enriched for dominant disease genes and to lesser extent recessive disease genes. As of January 2021, 65% of genes in the lowest LOEUF decile are yet to have an OMIM disease association (calculated using data from https://omim.org), highlighting thousands of high-probability candidate disease genes awaiting discovery of the associated phenotypes.

### Missense Constraint

The majority of coding variants of uncertain clinical significance are missense variants, as reported in ClinVar, a public database where diagnostic laboratories and researchers share variant classifications (i.e., pathogenic, benign, and uncertain significance) (Pérez-Palma et al., 2019). Similar to methods for assessing loss-of-function constraint, methods to identify missense constraint have emerged by comparing the observed vs. expected numbers of missense variants modeled on population data (Lek et al., 2016; Samocha et al., 2017; Havrilla et al., 2019; Hayeck et al., 2019; Pérez-Palma et al., 2020). However, missense constraint varies across a gene; for example, unstructured regions are often less constrained than important functional domains, which has necessitated the development of regional missense constraint models (Havrilla et al., 2019; Abramovs et al., 2020). Furthermore, clustering patterns of pathogenic missense variants vary depending on the inheritance pattern. Turner et al. (2015) showed that dominant missense variants cluster more than recessive variants. Therefore, testing for non-random clustering patterns may identify novel regions of interest across large sample sizes (Turner et al., 2015). The application of these metrics has aided in the discovery of new gene disorders, including a *de novo* missense variant in a constrained region of *GABRA2* responsible for an early-onset epileptic encephalopathy (MIM: 618557) (Orenstein et al., 2018).

### Model Organism Databases

Identifying the phenotypic effects of gene disruption may be possible using model organisms when there is enough conserved evolutionary function of the pathway/organ/system involving the gene of interest. Where the functional consequences of most human gene variants are yet to be established, model organism databases serve as a useful resource. Indeed, 58% of human genes have orthologs with disease-associated phenotypes reported in at least one model organism (Mungall et al., 2017). Although non-human models are not necessarily perfect proxies for human diseases, they can still serve as important biological models, particularly when data are aggregated across species.

### Monarch Initiative

The Monarch Initiative^4^ is an open-science, collaborative project that aims to integrate phenotype–genotype data from a variety of species and sources (Mungall et al., 2017). Its user-friendly web portal promotes rapid assessment of phenotypes of orthologs in organisms and other species. Researchers can query genes, phenotypes, and diseases to identify candidate disease genes. Exomiser (Smedley et al., 2015) and Genomiser (Smedley et al., 2016) have utilized the Monarch Initiative in their gene prediction algorithms, which have led to diagnoses in participants in the UDN including the aforementioned discovery that the disruption of *STIM1* results in the York platelet syndrome (MIM: 805070) (Bone et al., 2016).

### Mouse Knockout Databases

Several mouse model organism databases exist including the Mouse Genome Database (MGD) (Smith et al., 2017; Bult et al., 2019), the Knockout Mouse Project (KOMP) (Austin et al., 2004), and the International Mouse Phenotyping Consortium (IMPC) (Meehan et al., 2017; Muñoz-Fuentes et al., 2018). These projects are building comprehensive catalogs of mammalian gene function, genotype–phenotype associations, and detailed phenotype data from mouse knockouts of every protein-coding gene (Muñoz-Fuentes et al., 2018; Bult et al., 2019). By 2019, the IMPC has fully or partially phenotyped 5,861 mouse genes, a third of which are non-viable (Muñoz-Fuentes et al., 2018; Cacheiro et al., 2019). Data from IMPC have aided the discovery of many novel Mendelian phenotypes (Bowl et al., 2017; Moore et al., 2018; Rozman et al., 2018). That said, there is still much more to be gleaned from mouse data; of the >10,000 mouse genes linked to at least one non-lethal phenotype in a mutant strain in MGD, Bamshad et al. (2019) showed that human orthologs for 72% of those genes are yet to be associated with a Mendelian disorder, providing another rich data source for candidate genes awaiting discovery of the human Mendelian phenotype.

### Incomplete Penetrance

Identifying novel disease genes can be challenged by incomplete penetrance, that is to say, when a disease-causing variant does not always result in any clinical expression of the disease. If a novel candidate gene has been associated with a given phenotype, yet some or all alleles are incompletely penetrant, then it can be difficult to gather sufficient evidence for a new disease–gene association using traditional genetic evidence such as case observations and familial segregation. To mitigate this, larger cohorts that can support statistical association studies must be pursued. Furthermore, researchers are exploring how combinations of genomic variants such as oligogenic models or co-inherited protective alleles, environmental exposures, and mosaicism may impact the onset of Mendelian disorders (Gruber and Bogunovic, 2020). One such approach is to specifically identify individuals that are resilient to rare disease, despite harboring pathogenic variants (Chen et al., 2016). Another area of interest is how *cis*-regulatory variation may modify the penetrance of coding variants (Castel et al., 2018).

### Gene-to-Patient Approaches

In recent years, government funding has invested in national sequencing projects for rare disease (Turnbull et al., 2018; Posey et al., 2019). Despite 100,000 individuals with rare disease being sequenced in the United Kingdom as part of the Genomics England 100KGP, the diagnostic rates are similar to those reported elsewhere in the literature (Houses of Parliament, 2015; Turnbull et al., 2018). However, the scale of such datasets welcomes opportunities for new approaches to novel gene discovery.

**FIGURE 3:**
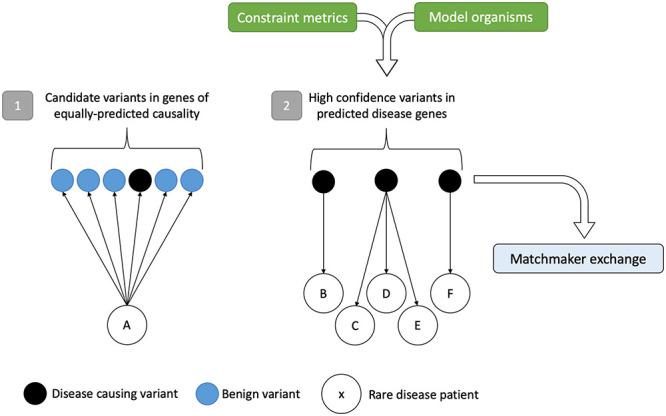
Gene-to-patient approach for improved rare disease diagnostics. Scenario (1) shows a traditional patient-to-gene approach. Following variant analysis, rare disease patient A has several potential disease candidates, of which one (in black) is the disease-causing variant hidden within the sea of benign variation. Without prior knowledge that any of these variants are causative, the only way to test their pathogenicity is by expensive functional studies on genes of equally predicted causality. In scenario (2), the approach is reversed. High-confidence disease-causing variants in genes identified by constraint metrics and model organism data can be matched to patients and compared to clinical phenotypes, circumventing the analytical noise precluding variant interpretation. In turn, this identifies the best candidates for follow-up and for data sharing in the MME. Variants/genes that match to more than one patient with the same or overlapping phenotypes can add credence to the method. Figure adapted from Seaby and Ennis (2020).

With increasing data available on genetic variation from a variety of sources including gene constraint, mouse models, and phenotype-driven methods, there is scope to utilize the power of large cohort sizes for novel gene discovery. Instead of bringing a patient to a gene, there are opportunities, with large enough sample sizes, to be sufficiently powered to detect rare variation and bring candidate genes to large genomic datasets from patients (Figure 3). These “gene-to-patient” approaches are already being applied to accelerate novel gene discovery and prioritize genes for functional studies (Seaby and Ennis, 2020).

### Integrating Multi-Omics Data

**FIGURE 4:**
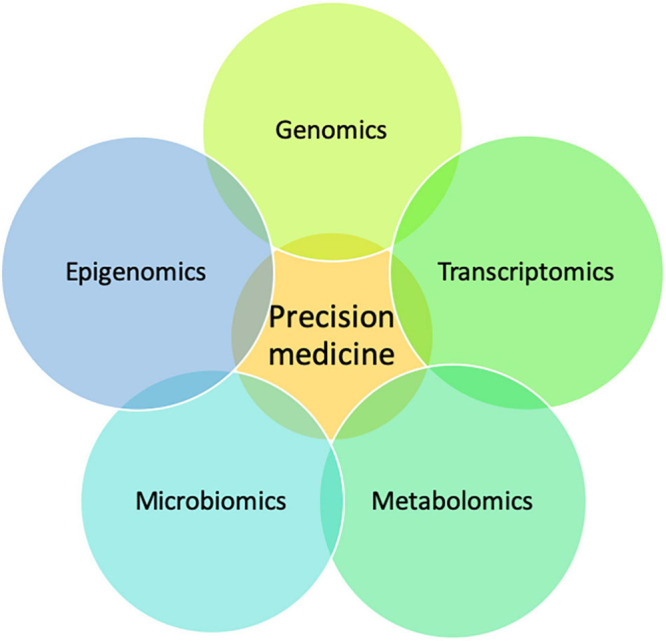
A multi-omics approach to precision medicine. Schematic showing how the integration of multi-omics data is complementary and important for precision medicine.

Multiple omics technologies such as epigenomics, transcriptomics, metabolomics, microbiomics, and proteomics are being adopted as approaches in the effort to delineate the functional impact of genetic variation (Figure 4) (Hasin et al., 2017). These integrative approaches can complement genomic data and aid in the validation and discovery of novel genes.

The Genotype-Tissue Expression (GTEx) project^5^ (Lonsdale et al., 2013) provides a public repository of tissue-specific gene expression and a multi-tissue reference for identifying variants associated with changes in gene expression or expression quantitative trait loci (eQTL) (Stranger et al., 2017). The GTEx consortium recently published results on their v8 release, providing insights into functional mechanisms and the architecture of genetic regulation (GTEx Consortium, 2020). The integration of transcriptome sequencing (RNA-seq) has led to improved diagnostic rates for Mendelian diseases (Kremer et al., 2017; Wai et al., 2020), even when no strong candidate variants were identified from exome or genome data (Cummings et al., 2017). Expression outliers, altered splicing, and allelic imbalance in the transcriptome due to non-sense-mediated decay can all be clues to candidate genes worth closer scrutiny in the exome or genome data (Brechtmann et al., 2018; Green et al., 2018; Mertes et al., 2021). Large-scale transcriptome data can also be used in network analysis (Deelen et al., 2019). One caveat with RNA-seq is that splicing aberrations and differential gene expression are best assessed by sampling disease-relevant tissues. However, these may not always be clinically accessible, e.g., brain tissue in neurodevelopmental disorders. Aicher et al. (2020) showed that many splicing events in non-clinically accessible tissues are lowly expressed and poorly evaluated from more commonly accessible tissues such as skin and blood. The authors developed a tool (MAJIQ-CAT) which allows researchers to explore potentially accessible tissues that best represent splicing in genes of interest (Aicher et al., 2020). A recent preprint in 2021 describes an alternative approach that has advantages over expression-based methods. Minimum Required Sequencing Depth (MRSD) informs biosample selection (whole blood, lymphoblastic cell lines, and skeletal muscle) by estimating the minimum sequencing depth required from RNA-sequencing to achieve desired coverage across a given gene or gene panel. The authors reported high precision, and their results suggest that lymphoblastic cell lines may be suitable for ∼70% of established disease gene panels (Rowlands et al., 2021).

The Encyclopedia of DNA Elements (ENCODE) and Roadmap Epigenomics projects have been instrumental in the generation of human reference epigenomes and epigenome maps, mainly from cell lines (Bernstein et al., 2010; Dunham et al., 2012; Satterlee et al., 2019). These data have successfully been used to conduct research on how the epigenome contributes to human development, environmental factors, and disease mechanisms (Dunham et al., 2012; Satterlee et al., 2019). More specifically, one of most commonly studied epigenetic phenomena, DNA methylation, is aiding diagnosis and gene discovery. Alterations in DNA methylation patterns are implicated in imprinting disorders and diseases of short tandem repeat (STR) expansions. The application of DNA methylation analyses has been successful in identifying molecular diagnoses in neurodevelopmental disorders where clinical microarray and other conventional genetic testing have been non-diagnostic (Aref-Eshghi et al., 2019; Turinsky et al., 2020). LaCroix et al. (2019) investigated cases of Baratela–Scott syndrome (BSS) (MIM: 615777) and identified hypermethylation of exon 1 of *XYLT1* associated with a GGC expansion and gene silencing. This not only confirmed BSS as a trinucleotide repeat expansion disorder but also highlighted the relative prevalence of methylation abnormalities in the disease pathogenesis of BSS. The hypermethylated allele accounted for 50% of the pathogenic alleles in their cohort, showcasing the importance of investigating epigenetic changes in disease cohorts with missing heritability (LaCroix et al., 2019).

National biobanks such as the United Kingdom Biobank (Bycroft et al., 2018), United States All of Us Research Program (Sankar and Parker, 2017), and Finland Biobank (FinnGen) provide opportunities to study genomic data and phenotype data alongside associated molecular markers from electronic medical records. While their data are best studied in the context of complex disease, they are also important in rare disease by providing population-level allele frequencies, biomarker results, and phenotypic information for comparative analyses. Unlu et al. (2020) utilized Vanderbilt’s biobank BioVU to identify a phenotypic profile that aided in the identification of a novel Mendelian syndrome CATIFA (cleft lip, cataract, tooth abnormality, intellectual disability, facial dysmorphism, attention-deficit hyperactivity disorder) that is due to loss of function of *RIC1* (MIM: 618761).

The emerging application of metabolomics with exome/genome sequencing is helping to improve diagnostic rates in rare disease. Targeted and untargeted metabolomics are proving successful in validating variants of uncertain significance in inborn errors of metabolism (Graham et al., 2018; Almontashiri et al., 2020). It is hoped that with increasing research, metabolomics will continue to complement rich genomic data and aid in discovery of novel genes.

These aforementioned approaches often applied in combination have been pivotal both in clinical diagnostics and in identification of novel candidate disease genes. For example, a study in 2015 using epigenomics, comparative genomics, and genome editing identified a pathway for adipocyte thermogenesis regulation involving *IRX3, IRX5*, and *ARID5B* in obesity (Claussnitzer et al., 2015), and in 2017, the complex I assembly factor *TIMMDC1* was established as a novel mitochondrial disease–gene by utilizing genomic and transcriptomic sequencing (Kremer et al., 2017).

## Discussion

The NGS era has undoubtedly accelerated novel Mendelian gene discovery for significant patient benefit. Although in recent years there has been increasing interest in the non-coding space, there is still much to be gleaned from the human exome (Bamshad et al., 2019; Posey et al., 2019). Furthermore, disease–gene associations are complex; phenotypic and functional consequences of variation across a gene are highly variable and influenced by variant type, inheritance pattern, and gene position. Following identification of the disease–gene relationship, characterization of the full allelic series is needed.

With many strategies now available for novel gene discovery, best practices are likely to benefit from the aggregation of methods. Additionally, as drives for data uniformity are developed and adopted (e.g., use of HPO terms, standardized disease nomenclature, functional equivalence variant calling pipelines, and joint calling data) (Regier et al., 2018), it will become far easier to automate bioinformatics pipelines that are capable of processing and integrating data from a variety of rich datasets to increase power for diagnosis and gene discovery.

### Role of Data Sharing

It is unquestionable that open science and data sharing have been pivotal in uplifting diagnoses and advancing the field of genomic medicine. International collaborations are now commonplace in matching patients across the globe with specific genotypes, leading to high-impact publications on novel gene discoveries (Philippakis et al., 2015; Azzariti and Hamosh, 2020). Furthermore, the use of variant databases such as ClinVar^6^ have proven invaluable in providing the scientific community with a repository of variants, classified by pathogenicity, that can be applied to variant analysis for diagnostic interpretation (Landrum et al., 2014).

The success of open data science has been further driven by cloud computing. The presence of large datasets on cloud platforms can facilitate the access of desired data within a secure data-sharing platform. Examples include NHGRI’s Genomic Data Science Analysis, Visualization, and Informatics Lab (AnVIL) Space^7^ where rare disease data from the Centers for Mendelian Genomics along with data from additional projects such as 1,000 Genomes, Centers for Common Disease Genomics, and GTEx can be accessed after application in dbGaP. Other trusted research environments include NHLBI’s BioData Catalyst cloud platform with TOPMed data; Genomics England Research environment where 100 KGP data are stored and accessed; and RD-Connect with rare disease genomic data from various European sources. In these trusted research environments, increasingly large amounts of data can be aggregated; researchers can bring tools directly to the data and share these analysis workflows, saving time, expense, and security risks of moving and maintaining local copies of large genomic datasets. However, data sharing presents its challenges. There is still urgent need for an international code of conduct that provides clear, unified data-sharing rules across jurisdictions that comply with regional laws such as the European General Data Protection Regulation (GDPR) and the United States’ Health insurance Portability and Accountability Act (Phillips et al., 2020).

### Addressing the Translational Gap

Our understanding of the genetic basis of rare disease is constantly changing with new genes and variation being linked to disease at a rapid pace. Given the direct application of these discoveries to the clinical diagnosis of rare disease in patients, guidance is needed for understanding what information is ready to be incorporated into clinical care and what mechanisms are needed to quickly translate that information into medical practice. The Clinical Genome Resource (ClinGen) has developed a systematic framework for evaluating genetic and functional evidence for disease–gene relationships, enabling their classification as definitive, strong, moderate limited, no human evidence, disputed, or refuted with respect to their reported role in disease (Strande et al., 2017). ClinGen supports Gene Curation Expert Panels that bring together international groups of disease and curation experts to evaluate gene–disease claims in their respective fields^8^. ClinGen’s efforts are combined with other public and private gene curation efforts and are accessible within the Gene Curation Coalition database^9^. Currently, it is recommended that a gene–disease relationship reach moderate classification before it is included on predefined diagnostic gene panels for specific conditions (Bean et al., 2020). However, when performing exome and genome approaches on individuals with rare disease, variation can be detected in genes that have not yet been linked to disease but may be strong candidates. Although practices vary between laboratories and countries, some professional standards recommend reporting these findings back to patients when there is a reasonable chance that new evidence may evolve over time to strengthen the gene–disease relationship, similar to the return of variants of uncertain significance in genes already linked to the patient’s condition (Richards et al., 2015; Rehder et al., 2021). This approach also allows patients to be partners in solving the causes of rare disease (Mnookin, 2014). It is hoped that such a framework will achieve global recognition and be universally adopted to ensure consistency in translating research findings into the clinic.

### Clinical Impact of Novel Mendelian Conditions

While novel gene discoveries widen the known functional repertoire of disease genes, the focus and drive are ultimately uplifting diagnosis rates and improving patient outcomes. There have been thousands of pivotal Mendelian discoveries throughout history, and each one is no more important than another, at least not for the families involved.

Since the discovery of the *CFTR* gene in 1989 (Kerem et al., 1989), we are now able to diagnose cystic fibrosis (MIM: 602421) rapidly, predict pancreatic functional status, and plan preventative care with modulator therapy (Ramsey et al., 2011; Farrell et al., 2020). In 2004, the discovery that hypermorphic or gain-of-function variants of *PCSK9* cause familial hypercholesterolemia type III (MIM: 603776) (Timms et al., 2004) has led to the successful development and FDA approval of monoclonal antibodies against PCSK9, which are also used to treat non-familial forms of hypercholesterolemia (Blom et al., 2014; Roth et al., 2014; Cannon et al., 2015; Kereiakes et al., 2015). As more collaborative, cohort-based studies have emerged in the NGS era, many candidate genes have been discovered that have directly impacted treatment and clinical outcomes. In one study on neurometabolic disorders, whole-exome sequencing diagnosed 68% of patients and identified 11 novel candidate genes, leading to a targeted intervention in 44% of patients (Tarailo-Graovac et al., 2016).

Diagnosing Mendelian disorders as a direct result of novel gene discovery not only impacts the primary patient involved but their families and caregivers. Families of children with rare genetic diseases are adversely impacted by lack of peer support groups and psychological support as well as delays in diagnosis (Anderson et al., 2013). Parents of children with rare disorders have called for better education, reduction in avoidable diagnostic delays, and early access to interventions and treatments (Zurynski et al., 2017). For many, a genetic diagnosis can be life changing, even in the absence of a therapeutic option (Lingen et al., 2016). Following diagnosis, quality of life is often improved by participation in support groups that can provide longitudinal prognostic information, genetic counseling, and informed reproductive decisions with opportunities for pre-implantation genetic diagnosis or prenatal testing particularly in the case of inherited variants where there is a sizable recurrence risk (Sexton et al., 2008; Wojcik et al., 2020).

### Financial Impact of Novel Discoveries

Undiagnosed rare diseases are hugely expensive. A typical patient’s diagnostic odyssey lasts an average of 8 years and costs a total of $5,000,000 throughout a patient’s lifetime (Chong et al., 2015). Two prospective Australian studies have shown that early exome sequencing is making significant headway as a cost-saving diagnostic approach. Stark et al. (2017) showed that integrating whole-exome sequencing as a first-line test had an incremental cost saving per additional diagnosis of (converted to United States dollars) $1,543 (95% CI: $92–4,143). The cost per diagnosis was $4,248 (95% CI: $3,425–5,588), $14,893 less than standard diagnostic care (Stark et al., 2017). Tan et al. (2017) concluded that whole-exome sequencing performed at initial presentation to tertiary care resulted in an incremental cost saving of (converted to United States dollars) $6,383 per additional diagnosis (95 CI: $3,045–10,900) compared with standard diagnostic care. However, cost savings are only possible when sequencing can identify the causal variant. Therefore, every new genetic disorder identified, published, and shared in publicly available databases will have wide-reaching diagnostic and cost-saving potential. Taking the average of costs saved per additional diagnosis from the two studies ($3,964) and extrapolating this on 100,000 patients could save an estimated US$400 million.

## Looking to the Future

It is estimated that by 2025, 60 million patients will have their genome sequenced in a research or healthcare setting (Birney et al., 2017). While the sheer volume of data poses computational challenges, it also provides opportunities to learn more about the genetic architecture of health and disease. However, this necessitates improved methods for interpreting the spectrum of functional variation across all genes and particularly in the interpretation of non-coding variation, an area of investigation still in its infancy but beginning to make headway. Indeed, disruption of non-coding topologically associated domains have been associated with limb malformations (Lupiáñez et al., 2015; Spielmann et al., 2018), and non-coding variants upstream of *PRDM13* and *CCNC* have been linked to North Carolina macular dystrophy (Small et al., 2016; Green et al., 2021). While efforts like the Atlas of Variant Effect Alliance are working toward achieving the mammoth goal of interpreting the impact of all genomic variation, there is still a long way to go (Matreyek et al., 2018; Jepsen et al., 2020). It is expected that as data pour in across a variety of species and sources, more and more methods will adopt machine learning and deep learning techniques to find patterns and disease associations, but the utility of these approaches is limited by the quality of the training data and other factors influencing data interpretation. For novel gene discovery, perhaps one of the most powerful resources would be to build a publicly available human knockout database that links naturally occurring null variants in genes and supportive functional evidence to shared human phenotype data. This is an exciting time for novel gene discovery—the end is by no means in sight.

## Author Contributions

ES wrote the first draft of the manuscript, performed full literature review, and conceived idea for review. HR made changes to subsequent drafts of the manuscript and conceived idea for review. AO’D-L made changes to subsequent drafts of the manuscript, contributed to literature review, and conceived idea for review. All authors contributed to the article and approved the submitted version.

## Conflict of Interest

The authors declare that the research was conducted in the absence of any commercial or financial relationships that could be construed as a potential conflict of interest.

## Footnotes

1 https://fabricgenomics.com/

2 https://www.emedgene.com/

3 https://gnomad.broadinstitute.org

4 https://monarchinitiative.org

5 https://gtexportal.org/home/

6 https://www.ncbi.nlm.nih.gov/clinvar/

7 https://anvilproject.org/data/

8 https://clinicalgenome.org/affiliation

9 https://thegencc.org
